# Audit of the Adhd Care Pathway Compliance

**DOI:** 10.1192/bjo.2026.11683

**Published:** 2026-06-30

**Authors:** Harleen Kaur, Bethan Brace

**Affiliations:** North Staffordshire combined healthcare NHS trust, Stoke on Trent, United Kingdom

## Abstract

**Aims:**

**Aim:**

To evaluate compliance with the local ADHD care pathway across eligibility, assessment, treatment, monitoring, and documentation standards, and to identify areas for improvement.

**Background:**

The audit was initiated to assess adherence to the local ADHD care pathway, ensuring consistent and standardised delivery of ADHD care in line with Trust policy. The Local Trust ADHD Pathway is based on NICE NG87 (2018) and includes standards for appropriate referral and inclusion criteria, holistic assessment and care planning, use of outcome measures (ReQOL, DIALOG, GBO), medication initiation and ESCA completion, as well as risk assessments, safety planning, and regular follow-up documentation. Adherence to these standards facilitates consistent, high-quality care and aligns with national gold standard guidance.

**Methods:**

This was a retrospective audit of patients managed within the local ADHD care pathway. The audit population comprised 79 patients, with one excluded due to disengagement prior to assessment, resulting in a final sample of 78. Inclusion criteria were active cases on the ADHD pathway, while exclusion criteria were patients who disengaged prior to assessment. Data were collected from electronic patient records on Lorenzo.

**Results:**

All patients met pathway eligibility criteria and were allocated to a qualified keyworker. Initial ReQoL was completed in 64% of cases (50/78). A holistic assessment was completed for 88% of patients (69/78). Care plans were completed for all patients (78/78); however, only 49% included DIALOG and Goal-Based Outcomes. Risk assessments were completed in 99% of cases (77/78), while safety plans were documented for 76% (59/78). Medication was initiated in 76 of 78 patients, with ESCA documentation sent for 62 patients; 14 had not yet stabilised, and four ESCA requests were rejected by the same GP practice. All patients received 6-monthly reviews, although documentation of discussions regarding deprescribing or medication holidays was limited to three cases.

**Conclusion:**

This audit demonstrated strong adherence to the local ADHD care pathway across referral, assessment, prescribing, and monitoring stages. High levels of compliance were observed for referral appropriateness, completion of assessments, medication prescribing, and structured follow-up. However, engagement with outcome measures was inconsistent, and variation in documentation practices limited standardisation. Limited documentation of discussions regarding deprescribing or medication holidays highlights an area for further clinical reflection and quality improvement.

